# Two cases of combined patellar tendon avulsion from the tibia and patella

**DOI:** 10.1051/sicotj/2018014

**Published:** 2018-05-23

**Authors:** Viachaslau Bradko, William T. Stoll, Lee S. Haruno, Scott B. Rosenfeld, Scott D. McKay

**Affiliations:** 1 Department of Orthopaedic Surgery, Baylor College of Medicine, Houston, TX USA; 2 Division of Orthopaedic Surgery, Texas Children's Hospital, Houston, TX USA

**Keywords:** Knee injuries, bifocal avulsion, patella, patella tendon, tibial tuberosity

## Abstract

Avulsion fractures of the inferior pole of the patella and proximal tibial apophysis are independently rare injuries. They occur in children due to the relative weakness of the apophyseal cartilage compared to the ligaments and tendons. The combination of these two fractures, is exceedingly rare, with only a few previously described cases in the literature. Due to the infrequent presentation of this injury, careful examination and consideration of advanced imaging is important for diagnosis and preoperative planning. Here we present two cases of combined sleeve fractures of the inferior pole of the patella and tibial apophysis, with discussion of the pathophysiology, classification, identification and management of the injury.

## Introduction

An intense muscle contraction may result in the rupture of a muscle or tendon, or an avulsion fracture. In contrast to adults, children are more likely to suffer from avulsion fractures due to the relative weakness of their apohyseal cartilage compared to connective tissues such as ligaments and tendons.

Fractures of the lower pole of the patella are relatively rare. As these are often predominantly cartilaginous avulsions, they are often difficult to distinguish on plain radiographs.

Avulsion of the proximal tibial apophysis is also a rare injury (<1 % of all apophyseal lesions [1]). It primarily affects male adolescents approaching skeletal maturity, usually occurring between 13 and 16 years of age.

Concurrent avulsion-type fractures of the inferior patellar pole and tibial tubercle are exceedingly rare with only a few previously described cases in the literature [2–5]. In the following case reports, we present two such cases of bipolar patella tendon sleeve avulsions. The purpose of this report is to improve awareness of this unique injury pattern. Furthermore, we suggest advanced imaging for optimal pre-operative planning in suspected cases to avoid misclassifying these as isolated (unipolar) injuries.

## Case description

### Case 1

**Figure 1:**
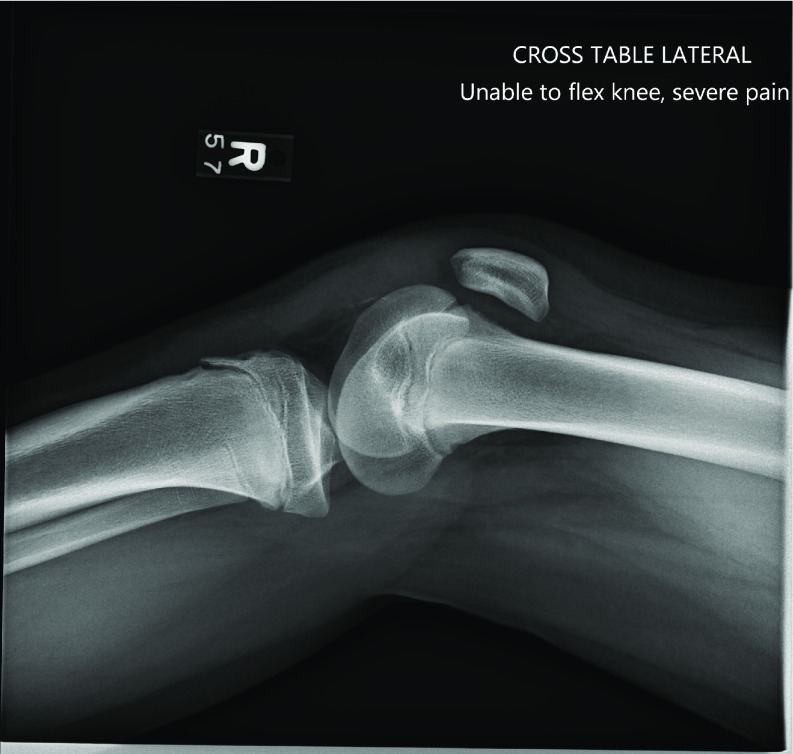
Lateral preoperative radiographs of the right knee for the patient described in Case 1. Patella alta is evident with possible patella sleeve fracture and accompanying avulsion of the tibial tubercle apophysis.

A 13-year-old male runner with history of contralateral (left) patellar dislocation developed pain and inability to bear weight in his right lower extremity following an awkward landing after jumping over a hurdle. On physical exam he was noted to have swelling and effusion of the affected knee with tenderness to palpation over the patella and tibial tubercle. Radiographs obtained in the emergency center demonstrated patella alta and a very small patella sleeve avulsion fracture (Figure 1). He was placed in a knee immobilizer. Operative management was performed the next day to restore the extensor mechanism.

**Figure 2:**
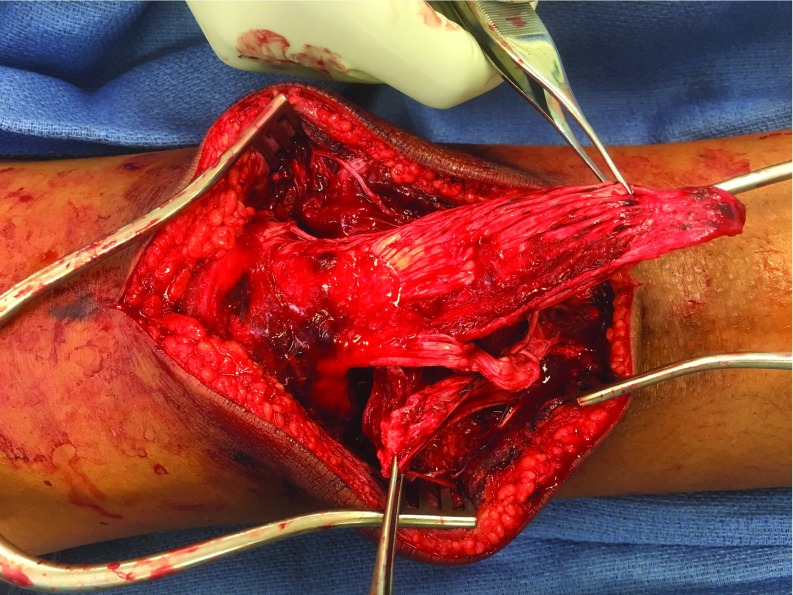
Intraoperative photograph of the right knee for the patient in Case 1, demonstrating bifocal avulsion of patella tendon.

**Figure 3:**
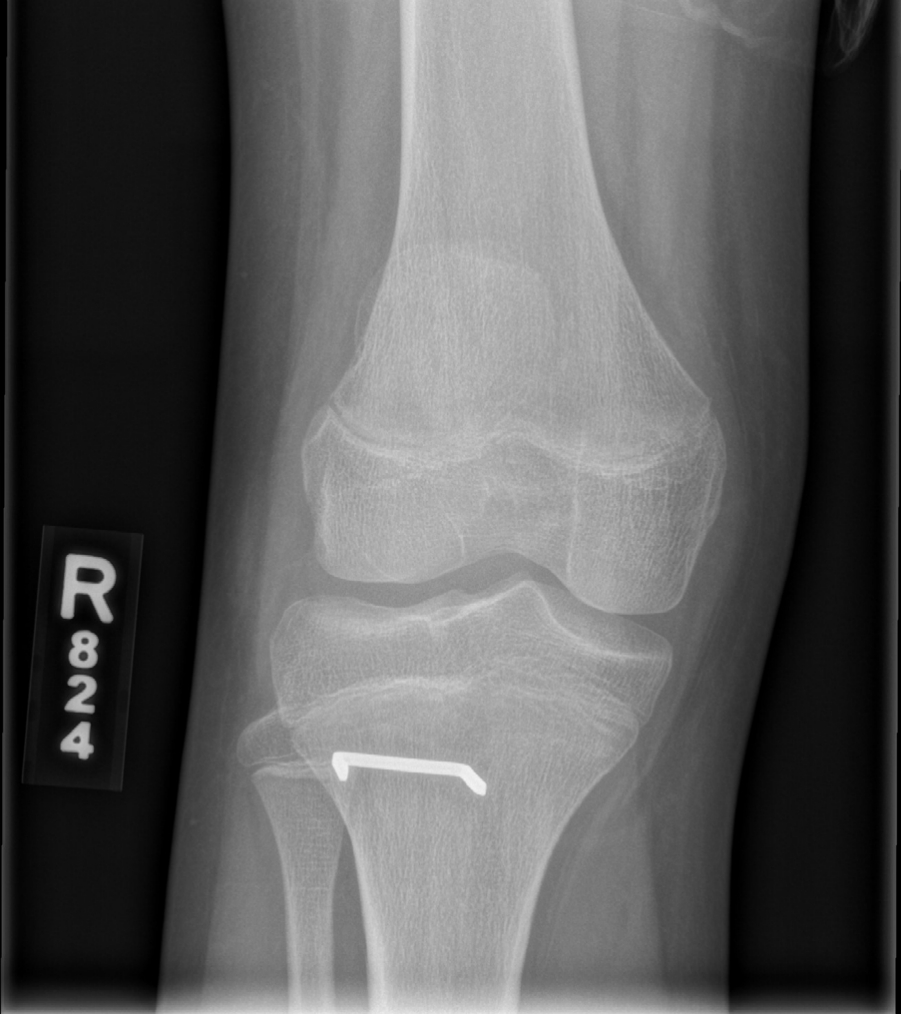
Anteroposterior postoperative radiographs of the patient described in Case 1. Surgical repair including staple fixation of the tibial tubercle avulsion fracture is evident.

**Figure 4:**
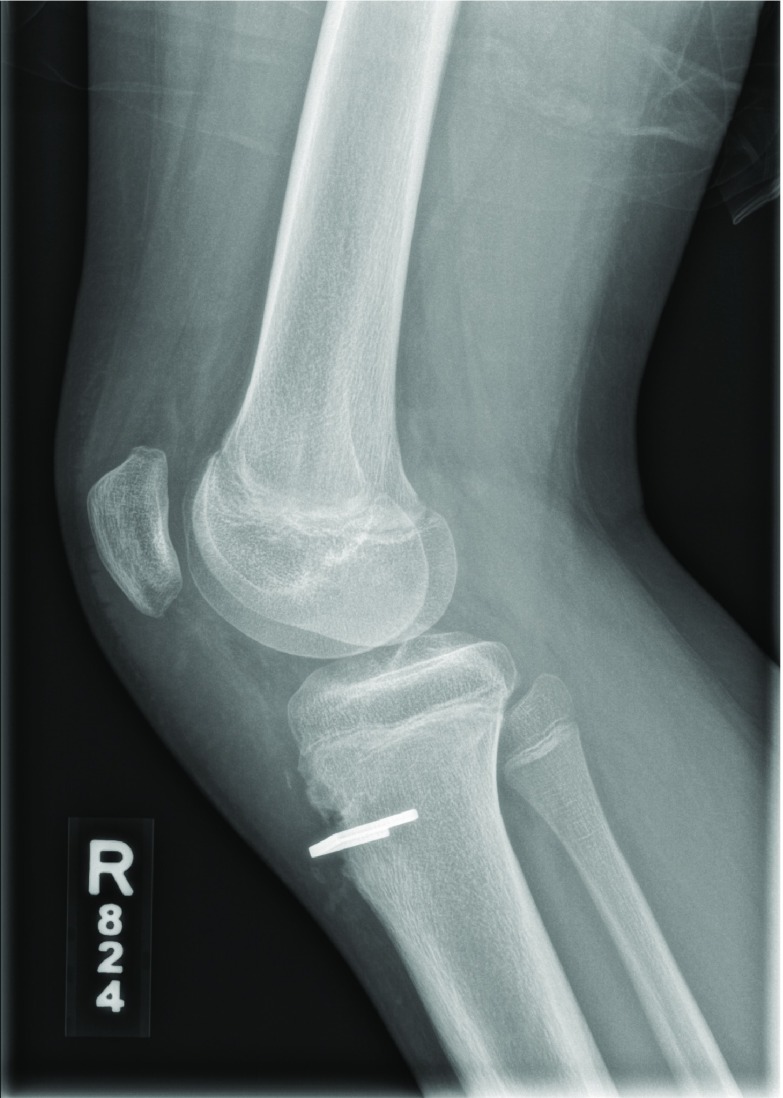
Lateral postoperative radiographs of the patient described in Case 1.

Intraoperative examination revealed an apophyseal avulsion of the medial 2/3 of the patella tendon from the tibial tubercle and avulsion of the lateral 1/3 of the tendon from the patella (Figure 2). Repair of the proximal avulsion was performed using #2 non-absorable suture in a Krakow type pattern to interlock the patellar tendon along each edge of the larger tendon pieces ending in the apophysis. This suture was then run through two bone tunnels in the patella to secure the apophysis in its original location. A second non-absorbable suture was also used in a Krakow pattern to repair the split between the medial 2/3 and lateral 1/3 of the tendon. The tibial tubercle apophysis repair was then secured with a 25 × 20 mm staple through the avulsed apophyseal fragment (Figures 3 and 4).

Postoperatively, the patient was placed in a knee immobilizer and with touch down weight bearing precautions and crutches for the initial four weeks following surgery. Follow-up six weeks after surgery demonstrated radiographic healing. Full range of motion had been achieved at his 3 months follow up.

### Case 2

A 12-year-old male athlete with history of Osgood-Schlatter disease developed acute pain in his left knee while jumping during a basketball game. He was unable to ambulate following the injury and was originally seen at an outside hospital, where he was treated for a presumptive patellar dislocation. Two attempts at reduction under sedation were unsuccessful, and the patient was transferred to the authors’ institution for further management. Examination at this time revealed patella alta and diffuse swelling. No neurovascular deficits were noted.

**Figure 5:**
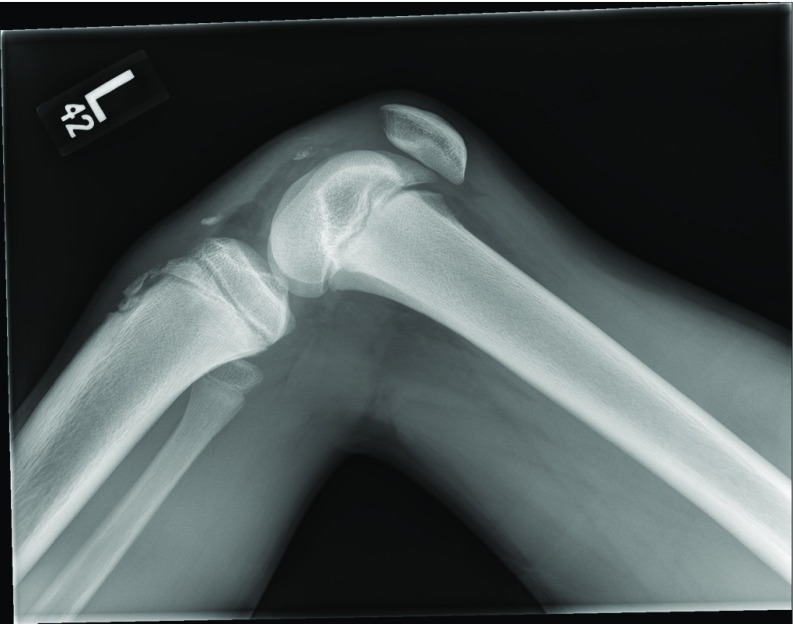
Lateral preoperative radiographs of the patient described in case 2. There is evidence of a displaced sleeve fracture from distal patella and a simultaneous avulsion from the tibial tuberosity accompanied by small bony fragments.

**Figure 6:**
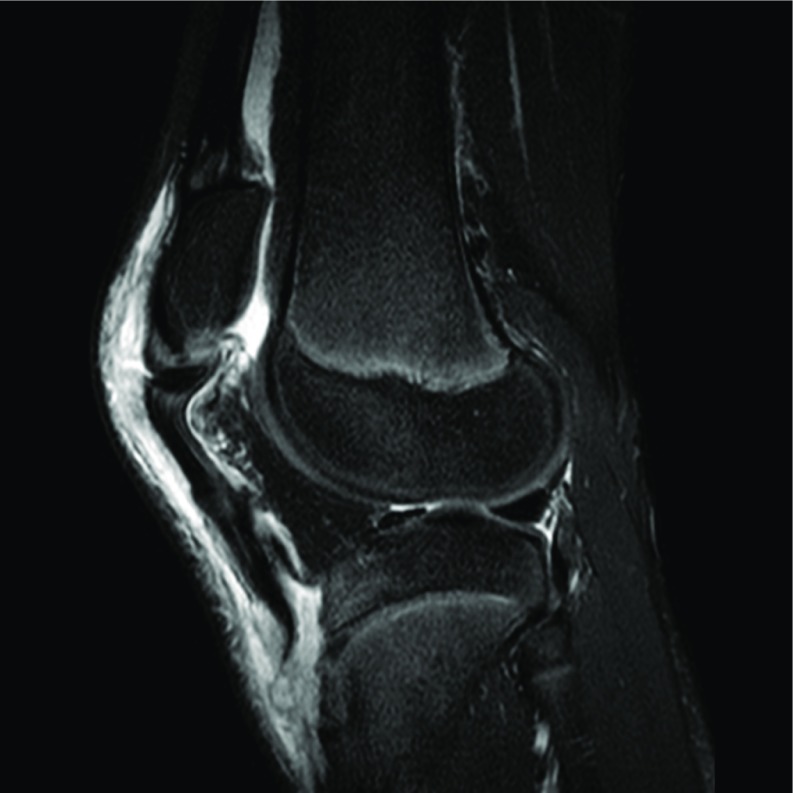
Preoperative sagittal MRI demonstrating bifocal avulsion sleeve fracture from both distal patella and tibial tubercle as described in Case 2.

**Figure 7:**
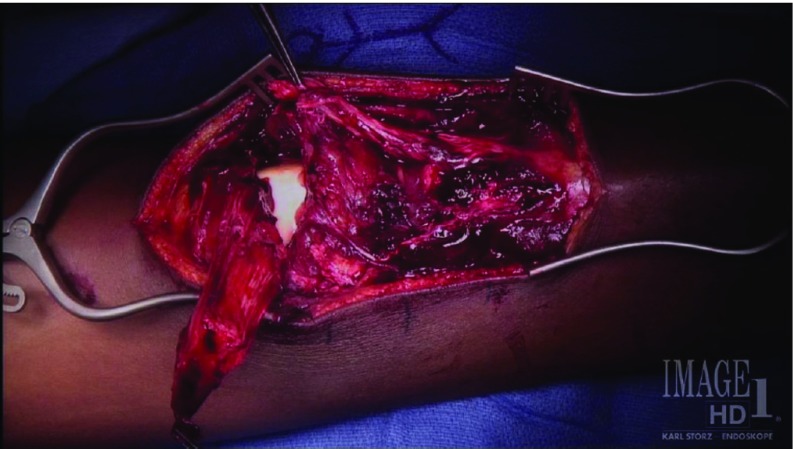
Intraoperative images for the patient in Case 2 depicting bipolar proximal and distal avulsions of the patella tendon.

Radiographs showed a likely inferior patellar sleeve fracture with a possible concomitant tibial tubercle fracture. MRI confirmed avulsion fractures of both the patellar and tibial insertions of the patellar tendon, as well as a quadriceps strain at the patellar insertion and tears of the medial and lateral patellar retinaculum (Figures 5–7). Operative repair of the proximal and distal patellar tendon avulsions was undertaken.

Intraoperative findings revealed a 75% avulsion of the tendon from its attachment at the medial tibial tubercle with the remaining lateral quarter of the tendon intact distally but detached from the inferior pole of the patella. Additionally, the fat pad and anterior capsule had also avulsed from the inferior aspect of the patella. The injury extended into the medial and lateral retinaculum at the level of the inferior patella. Repair of the patellar tendon at the distal avulsion site was performed with screw and ligament staple through the apophysis, and reinforced with Krackow sutures tied over a bone bridge in the anterior tibia. The proximal avulsion fracture was repaired with #2 non-absorbable sutures running from the tendon through patellar bone tunnels, and tied over the bone bridge at the superior patella. The retinaculum was repaired with absorbable suture.

**Figure 8:**
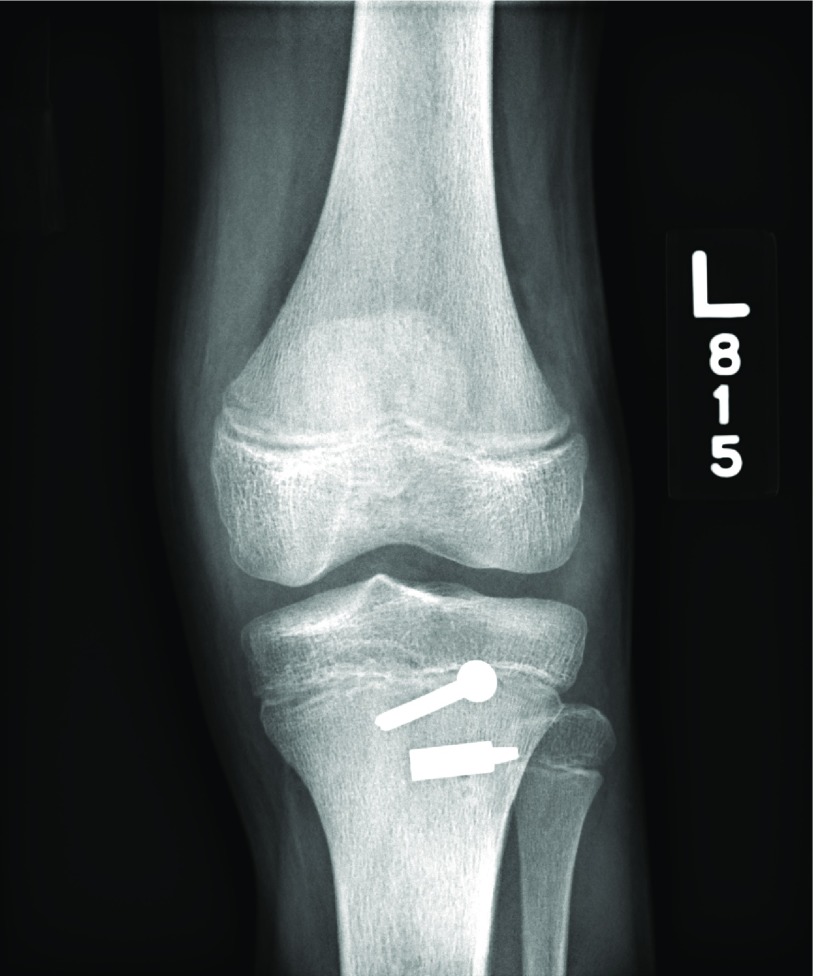
Anteroposterior postoperative radiographs of the patient described in Case 2.

**Figure 9:**
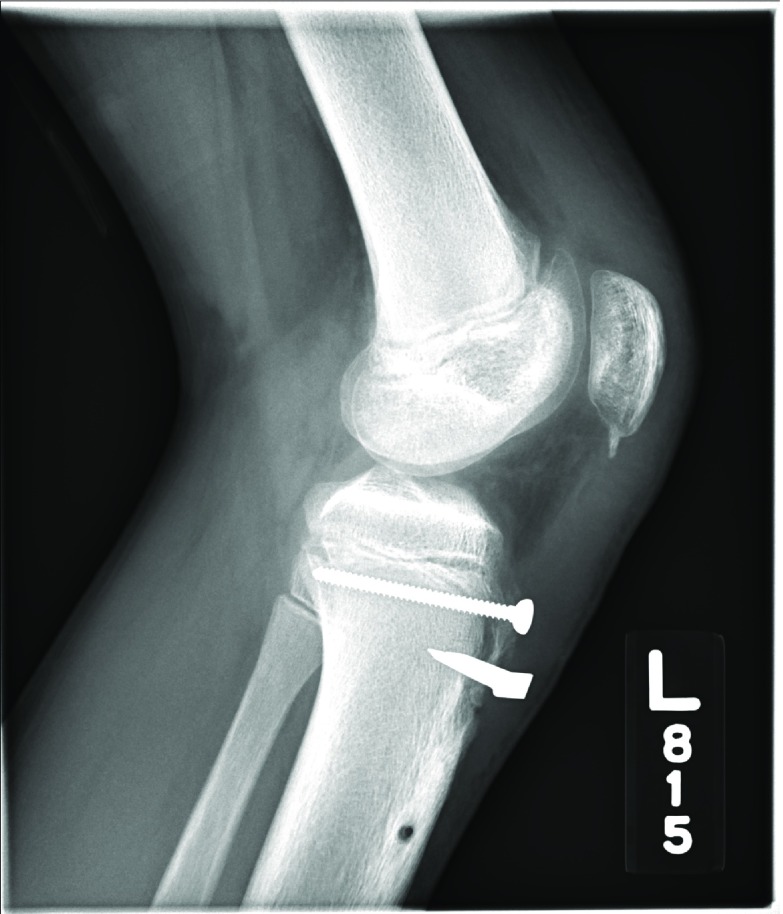
Postoperative radiographs of the patient described in Case 2. Surgical repair including screw and staple fixation of the tibial tubercle avulsion fracture is evident.

Postoperatively the patient was placed in a locked straight leg brace for one week (Figures 8 and 9). Following this initial week of recovery he began range of motion exercises and progression of weight bearing with physical therapy. At six weeks postoperatively he was tolerating full weight-bearing activity with the assistance of his brace. He was noted to have full range of motion in affected knee in 3 months follow up, when he was allowed to return to sport-specific activities in preparation for return to sport.

## Discussion

The occurrence of bifocal patellar tendon avulsion injuries in the absence of definitive radiographic signs was an especially important feature of these two cases. In Case 1, the diagnosis was not suspected from radiographs, and instead discovered intraoperatively. In Case 2, the diagnosis was suspected and then confirmed by MRI.

Tibial tubercle fractures in adolescent patients are typically avulsion-type injuries, given the relative weakness of the open physis compared to the superior strength of the patellar tendon at that stage of development. The classification of these injuries was originally proposed by Watson-Jones in 1955 and described three types of fractures spanning the tubercle, physis and knee joint. Type I involved an avulsion of the tubercle distal to the physis, while Type II involved the physis but spared the knee joint, and Type III extended into the knee joint itself. This classification system was later expanded on by Ogden and colleagues in 1980 to further characterize the fracture patterns associated with each injury, and propose specific treatment approaches. With his case report of tubercle fractures associated with associated patellar ligament avulsions, Frankl et al. suggested a Type C classification to the modified Ogden system [2]. Mosier and Stanitski observed this type of fracture in 2 cases of tibial tubercle avulsion fractures in a study reviewing 19 of these fractures in 18 patients [1]. Many of the documented cases of these bipolar fractures occur in association with a jump, as seen with our patients, with basketball or track as two of the most commonly associated sports [3–5]. Our two cases are consistent with the jumping mechanism, and provide two examples of operative management of these injuries (Table 1).

**Table 1:** Summary of reported cases including present case.

	Case	(Ref no.)	Age	Sex	Sports	Condition	Treatment	Materials
1	Clarke et al., 2016	[3]	16	Male	Football (soccer)	Was tackled while taking a shot	Internal fixation	Three lag screws and Krakow sutures
2	Stepanovich et al., 2016	[4]	12	Male	Skateboard	Fall	Internal fixation	Three cannulated screws and sutures
3	Hermansen et al., 2015	[5]	12	Male	Skateboard	Fall	Internal fixation	Soft anchor with sutures and metal-wire cerclage
4	Bradko et al., present article 2018		13	Male	Running	Awkward landing after jump	Internal fixation	Staple and Krakow sutures
5	Bradko et al., present article 2018		12	Male	Basketball	Jumping	Internal fixation	Screw, staple and Krackow sutures

Two traction apophysitis diseases postulated to be associated with avulsion-style fractures are Osgood-Schlatter Syndrome (OSS), at the tibial tubercle, and Sinding-Larsen Johansson (SLJ) Syndrome, at the inferior pole of the patella, respectively. Of the two, OSS is the more common and is due to repetitive traction on the secondary ossification center of the tibial tuberosity. It is most commonly seen in male athletes between 10 and 15 years of age, concurrent with the rapid growth seen in adolescents during this time. SLJ Syndrome is a rare but similar disease to OSS where the inferior pole of the patella at the site of the proximal attachment of the patellar tendon is affected. This condition is frequently seen in a younger cohort than the Osgood-Schlatter patients, most commonly at ages 10–12. Preceding pain is common for this kind of patients. Nevertheless, despite the fact of remote history of OSS in Case 1, neither of our patients had immediately preceding anterior knee pain before the described trauma.

## Conclusion

We report two cases of combined sleeve fractures of the inferior pole of the patella and tibial apophysis. In first case, preoperative images were confusing and the full complexity of this trauma were verified only during surgery. In the second case we used MRI examination preoperatively, which allowed us to fully characterize the origin of trauma pattern and to prepare proper option for its operative treatment. In our conviction patients of this age and with this type of trauma need extra attention during examination and preoperative planning, we suggest MRI study as clarifying imaging modality when radiographs are not conclusive.

## Conflict of interest

The authors declare that they have no conflict of interest.

## References

[1] Mosier SM, Stanitski CL (2004) Acute tibial tubercle avulsion fractures. J Pediatr Orthop 24(2), 181–184. 15076604 10.1097/00004694-200403000-00009

[2] Frankl U, Wasilewski SA, Healy WL (1990) Avulsion fracture of the tibial tubercle with avulsion of the patellar ligament. Report of two cases. J Bone Joint Surg Am 72(9), 1411–1413. 2229123

[3] Clarke DO, Franklin SA, Wright DE (2016) Avulsion fracture of the tibial tubercle associated with patellar tendon avulsion. Orthopedics 39(3), e561–e564. 27088354 10.3928/01477447-20160414-02

[4] Stepanovich MT, Slakey JB (2016) Combined tibial tubercle avulsion fracture and patellar avulsion fracture: an unusual variant in an adolescent patient. Am J Orthop 45(1), E31–E34. 26761925

[5] Hermansen LL, Freund KG (2016) Bifocal osseous avulsion of the patellar tendon from the distal patella and tibial tuberosity in a child. Knee Surg Sports Traumatol Arthrosc 24(3), 712–714. 26410095 10.1007/s00167-015-3800-8

